# Impact of altitude correction on bronchopulmonary dysplasia prevalence: A systematic review and meta-analysis

**DOI:** 10.1371/journal.pone.0322204

**Published:** 2025-04-22

**Authors:** Madina Syzdykova, Marina Morenko, Kseniya Shnaider, Saltanat Urazova, Ulbossyn Saltabayeva, Nelli Bugayeva, Zhuldyzay Kagenova

**Affiliations:** 1 Department of Children’s Diseases with courses in Allergology, Immunology, Hematology and Endocrinology, NJSC “Astana Medical University”, Astana, Kazakhstan; 2 Department of Family Medicine, NJSC “Astana Medical University”, Astana, Kazakhstan; 3 Department of Nursing, NJSC “Astana Medical University”, Astana, Kazakhstan; Pennsylvania State University College of Medicine: Penn State College of Medicine, UNITED STATES OF AMERICA

## Abstract

Bronchopulmonary dysplasia (BPD) presents a significant respiratory challenge in infants born prematurely. Socioeconomic factors and environmental determinants, including altitude, play pivotal roles in shaping respiratory health outcomes among premature infants. This study aimed to investigate the prevalence of BPD among preterm infants based on altitude, considering the impact of altitude correction on prevalence estimates. By examining altitude-related variations in BPD prevalence, the study sought to provide insights essential for guiding interventions aimed at preventing and managing respiratory conditions in this vulnerable population. The study protocol was registered with PROSPERO, and a systematic search of five databases (PubMed, Web of Science, ScienceDirect, ProQuest, and Google Scholar) was conducted without any restrictions on the date of publication. Eligible studies were identified based on predefined inclusion criteria, including retrospective or prospective studies reporting BPD prevalence at different altitudes, the use of standard diagnostic criteria for BPD, and the exclusion of studies involving non-human subjects or those lacking altitude-adjusted data. The risk of bias assessment was conducted using the Critical Appraisal Skills Programme checklist. Statistical analysis included calculating pooled prevalence estimates using a random-effects model, performing subgroup analyses, and assessing heterogeneity and publication bias. The search yielded 339 records, of which ten articles met the inclusion criteria and had a low risk of bias. The altitude-unadjusted BPD prevalence was 41.35% (95% CI 28.62; 55.34%) and ranged from 19.73% (95% CI 16.44; 23.48%) to 71.02% (95% CI 68.33; 73.56%) across different altitude categories. The altitude-adjusted pooled mean BPD prevalence was 26.70% (95% CI 19.60; 35.25%). This systematic review and meta-analysis highlight altitude-related variations in BPD prevalence among preterm infants. Altitude adjustment is crucial for understanding the true prevalence of BPD and guiding tailored interventions in high-altitude regions.

## Introduction

Bronchopulmonary dysplasia (BPD) is a chronic lung disease that arises as a complication of mechanical ventilation and oxygen therapy administered to prematurely born infants experiencing respiratory distress [1,2]. Its impact is substantial, contributing significantly to the burden of chronic respiratory diseases among infants worldwide [3]. While many infants with BPD improve as they grow older, some may experience long-term respiratory complications [4]. These complications can include persistent respiratory symptoms such as wheezing, coughing, and shortness of breath, as well as an increased risk of respiratory infections [5]. Additionally, children with BPD may be more prone to developing asthma and other chronic respiratory conditions later in life. [5–7] Long-term outcomes in BPD highlight the importance of continued monitoring and support for respiratory health to mitigate the potential impact on quality of life.

The definition of BPD has evolved over time, reflecting advancements in neonatal care and a deeper understanding of the disease. Initially described by Northway et al. in 1967, BPD was characterized by severe lung injury in preterm infants exposed to mechanical ventilation [8]. Modern definitions, such as those proposed by the National Institute of Child Health and Human Development (NICHD) and the Vermont Oxford Network (VON), focus on the need for supplemental oxygen or respiratory support at 36 weeks postmenstrual age (PMA) [9,10]. These evolving definitions have implications for reported prevalence rates, as diagnostic criteria and severity classifications vary across studies and regions. This variability highlights the need for standardized approaches to defining and reporting BPD, particularly in the context of environmental factors such as altitude.

Socioeconomic status and environmental factors play crucial roles in determining long-term outcomes for individuals with BPD [11,12]. Socioeconomic disparities can hinder access to quality healthcare, nutritional support, and adequate housing conditions, all of which significantly influence the severity and management of BPD [11,12]. Moreover, numerous studies have investigated the impact of environmental factors, such as altitude, on respiratory conditions [13–15]. As altitude increases, both barometric pressure and the partial pressure of inspired oxygen (PiO2) decrease [16]. According to the alveolar gas equation, this decrease in PiO2 results in lower alveolar oxygen levels, necessitating higher fractions of inspired oxygen (FiO2) to maintain adequate oxygenation [16,17]. This physiological adaptation is particularly relevant for preterm infants, who often require supplemental oxygen to mimic sea-level conditions. Failure to adjust for altitude may result in an overestimation of BPD prevalence, particularly in high-altitude regions [18,19].

The relationship between altitude and BPD is further supported by the hypothesis that, at higher altitudes, an increased FiO2 is required to achieve the same oxygenation levels as at sea level. Based on the NICHD definition of BPD, which includes the need for oxygen support at 36 weeks PMA, this requirement may lead to a higher prevalence of BPD in high-altitude regions [8]. Moreover, altitude may contribute to variations in BPD severity across geographical areas, as the physiological stress of hypobaric hypoxia can exacerbate lung injury and impair alveolar development in preterm infants [20,21].

The pathophysiology of BPD involves a complex interplay of genetic, environmental, and iatrogenic factors. Altitude contributes to this pathophysiology by exposing preterm infants to chronic hypobaric hypoxia, which can disrupt normal lung development and increase oxidative stress. These effects may exacerbate the alveolar simplification and vascular dysregulation characteristic of BPD, leading to more severe disease in high-altitude populations [22,23]. Understanding these mechanisms is critical for developing targeted interventions to mitigate the impact of altitude on BPD outcomes.

Despite the demonstrated importance of reporting altitude-adjusted BPD rates in various studies, a comprehensive meta-analysis systematically examining BPD prevalence based on altitude and altitude correction is currently lacking. Therefore, the primary objective of this study is to systematically review the literature and conduct a meta-analysis to investigate both the unadjusted and adjusted prevalence of BPD among preterm infants, defined as those born at or before 32 weeks of gestation or weighing less than 1500 grams at birth, based on altitude.

## Materials and methods

Ethical review and approval were not required for this study since it is a systematic review of existing published literature. The study protocol is registered with the PROSPERO International prospective register of systematic reviews CRD42024542609 [9].

### Search strategy

The PROSPERO database was searched to identify the registration of similar studies, and no similar studies were found. We conducted a subsequent search in four major electronic literature databases: PubMed, Web of Science, ScienceDirect, and ProQuest. Additionally, Google Scholar was searched to capture relevant studies that might not be indexed in traditional literature databases. In Google Scholar, the search was restricted to titles only to enhance relevance and manage the volume of results. Studies from all years were considered, and the final literature search was conducted on March 9, 2024. The search strategy included the following keywords: “bronchopulmonary dysplasia”; “BPD”; “altitude”; and “prevalence”.

### Eligibility criteria

Methodologically, the literature screening and synthesis followed the recommendations of Preferred Reporting Items for Systematic Reviews and Meta-Analyses (PRISMA) [10]. The full PRISMA checklist for systematic reviews is provided in S1 Table. PECOS inclusion criteria for the studies: Population (P): 1) Preterm infants born at less than 33 weeks of gestation or with a very low birth weight (VLBW) of less than 1500 grams at birth. Exposure (E): Studies that included data on the altitude of the location where the infants were born. Comparison (C): BPD prevalence with altitude correction versus BPD prevalence without altitude correction. Outcome (O): Prevalence of BPD among the study population, as defined by supplemental oxygen dependency at 36 weeks postmenstrual age (PMA). Study design (S): Retrospective or prospective studies published in peer-reviewed journals in the English language. Exclusion Criteria: 1) Studies lacking sufficient data on BPD prevalence, altitude, or altitude correction methodologies. 2) Studies solely focusing on VLBW infants with no emphasis on BPD prevalence. 3) Reviews, commentaries, and editorials. 4) Multiple studies from the same cohort with overlapping data; in such cases, the most comprehensive or recent publication was prioritized.

### Selection of studies and data extraction

The identified publications underwent deduplication, followed by a two-stage screening process. First, two independent researchers [M.S. and Z.K.] screened titles and abstracts to determine relevance based on predefined inclusion and exclusion criteria. Publications that met the eligibility criteria proceeded to full-text review. During this stage, the same two researchers [M.S. and Z.K.] independently assessed the full texts for eligibility. Any discrepancies between the reviewers were resolved through discussion, and if necessary, a third researcher [M.M.] was consulted to reach a consensus.

Following study selection, data extraction was performed using a standardized template developed through discussion and agreement among all authors. In adherence to PRISMA guidelines, two independent researchers [M.S. and Z.K.] extracted data separately to ensure accuracy and minimize bias. Any disagreements were resolved through discussion and evaluation by the third author [M.M]. The following data were extracted: first author, year, country, study design, BPD definition, gestational age or weight of the infants, altitude correction (yes, no, no information), number of total infants in the study, number of BPD patients, altitude, number of BPD patients after altitude correction.

### Risk of bias

We used the Critical Appraisal Skills Programme (CASP) Qualitative Research Checklist to assess the methodological quality of the studies included in our analysis [17]. This checklist comprised ten questions covering various aspects such as study objectives, methodology, design, recruitment, data collection, ethical considerations, analysis, findings, and overall value. Each criterion was rated as ‘yes’ if adequately described (scored as 1), ‘no’ if absent (scored as 0), and ‘can’t tell’ if unclear or incomplete (scored as 0.5). Total scores ranged from 0 to 10, with a score of at least 7 indicating satisfactory quality. Two researchers responsible for data collection [M.S. and Z.K.] independently assessed the risk of bias in the included studies. Any disagreements were resolved through discussion with the third author [M.M.].

### Statistical analysis

Using a random-effects model in R (version 4.3.2, 2023-10-31) within RStudio (version 2024.12.1+563), we employed the ‘meta’ and ‘metafor’ packages [20] to compute [20]the pooled mean prevalence of BPD along with 95% confidence intervals (95% CI). To visually represent the pooled estimates, forest plots were employed [21]. Heterogeneity among studies was assessed using the I²-statistic [22]. Sensitivity analysis which included the leave-one-out analysis was performed to identify studies significantly impacting the pooled prevalence estimates [21]. Subgroup analysis was carried out to investigate sources of heterogeneity. Studies were stratified into four altitude categories: sea level to less than 400 meters; 400 – less than 1000 meters; 1000–2000 meters; and 2000 meters and above. Publication bias was assessed using a funnel plot and Egger’s test, which examined potential asymmetry in the distribution of study results [21]. These assessments were conducted only when at least 10 studies were available.

### Certainty of evidence assessment

Certainty of evidence was evaluated using the Grading of Recommendations Assessment, Development, and Evaluation (GRADE) framework, as recommended by the Cochrane Handbook for Systematic Reviews of Interventions [23]. The assessment included the following domains: Risk of Bias, evaluated using the CASP checklist. Inconsistency, determined by the I² statistic, where I² > 75% was classified as “Serious,” I² > 50% as “Moderate,” and I² > 25% as “Not serious”. Indirectness, assessed based on PECOS criteria. Imprecision, determined by whether the 95% confidence interval (CI) of the pooled estimate crossed the minimally clinically important difference. Specifically, this criterion evaluates if the upper and lower bounds of the CI could lead to different clinical decisions [24]. Publication Bias, evaluated using the funnel plot and Egger’s test, which require a minimum of 10 studies. The assessment followed the approach outlined in research notes on GRADE evaluation in systematic reviews [25].

## Results

**Fig 1:**
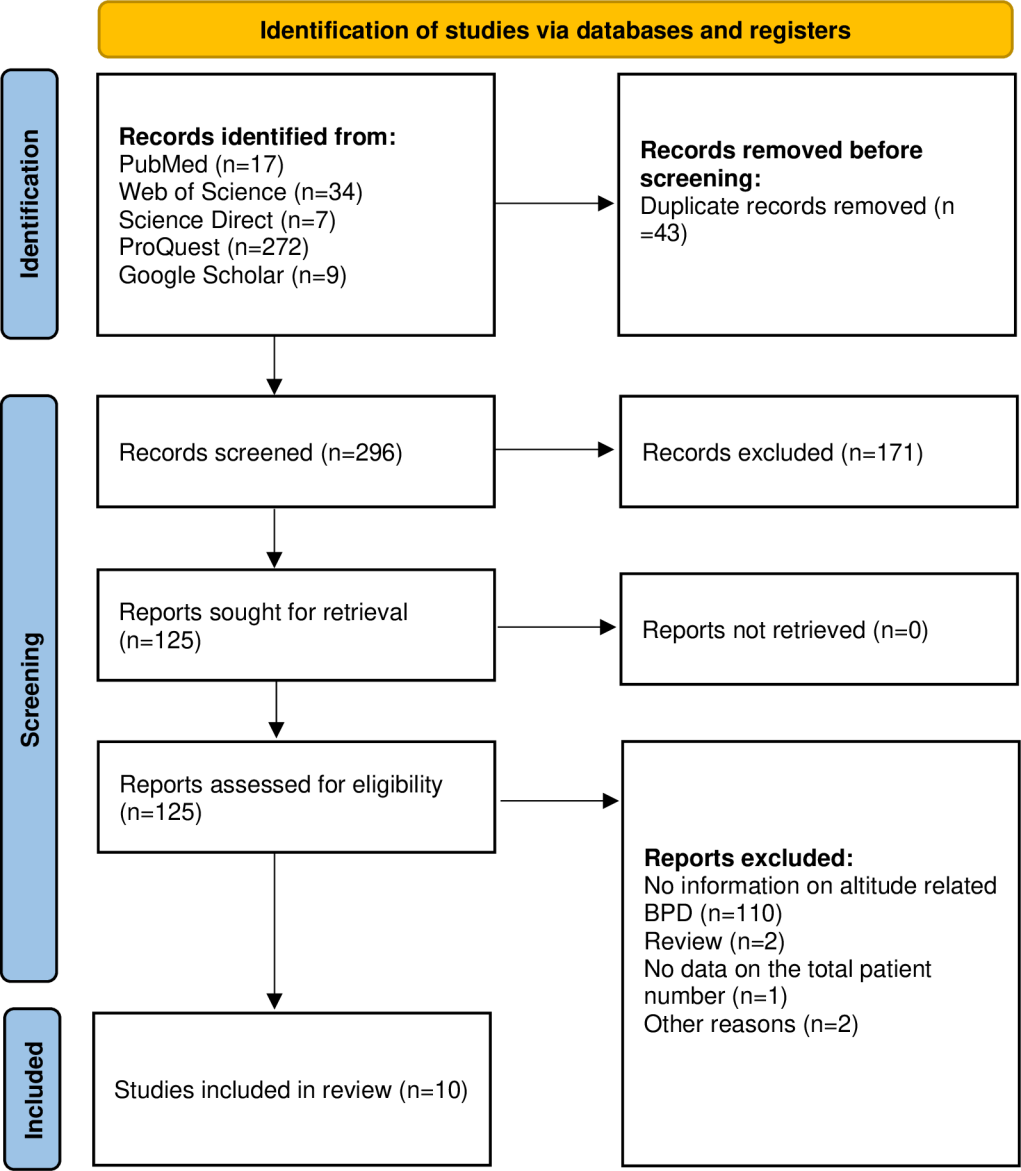
PRISMA flow chart of study selection [ 10].

An extensive search across PubMed, Web of Science, ScienceDirect, ProQuest, and Google Scholar databases identified 339 records. After removing duplicates, 296 unique records remained, of which 124 full-text articles underwent evaluation. Notably, one study examined a cohort of 11,953 oxygen-dependent infants sampled between 1998 and 2021 in Bogota, Colombia, a city located 2600 meters above sea level. This study reported BPD prevalence ranging from 2% to 20% among infants. However, due to the lack of data on the total number of newborns assessed in each year group, it was excluded from the final analysis [26]. Ultimately, ten articles met the inclusion criteria for the systematic review and meta-analysis. The study selection process is shown in Fig 1, with detailed results in S2 Table [10].

### Description of included studies and subjects

Table 1presents the study design and characteristics of patients. All studies were published between 2012 and 2022, with diverse geographical origins. Three studies originated from Colombia, three from the United States, one from Canada, and one from Saudi Arabia. Additionally, one study compared BPD rates between infants in China and Tibet, while another included multiple cities and countries from the Siben network. This review encompassed studies with diverse methodologies: seven cross-sectional studies, two case-control studies, one observational longitudinal study, and one cohort study. Notably, one study lacked criteria for BPD diagnosis [27]. In total, ten studies encompassed 11,192 preterm infants (mean sample size = 746 patients, range = 88–6011 patients). We excluded infants with a BPD rate at 28 days post-delivery and only included the BPD rates at 36 corrected weeks from the study by Buendia and coauthors [18]. Among the studies, three compared high altitude BPD rates to sea level or less than 400 meters altitude BPD rates, while two compared high altitude (more than 2000 meters) BPD rates to 400–1000 meters altitude BPD rates. Fourteen groups were included in the nine studies presenting altitude-unadjusted BPD prevalence rates, while six studies presented altitude-adjusted BPD rates. Notably, one study found no change in BPD prevalence rates after altitude adjustment at sea level, hence this group was excluded from the adjusted BPD prevalence meta-analysis [19]. Comprehensive data extraction details are presented in S3 Table.

**Table 1:** Summary of included articles.

First author, year	Country	Region	Study design	BPD definition	Gestational age or weight	Total sample size	Altitude (meters)	BPD prevalence reported
Britton, 2012 [28]	USA, Colorado Denver	North America	Cross-sectional	The Vermont Oxford Network (VON) criteria: a supplemental oxygen dependency at 36 weeks PMA.	<30 weeks GA or 1500 gr	131	1609 meters	Adjusted and unadjusted
Lee, 2012 [29]	Canada	North America	Cross-sectional	A supplemental oxygen dependency at 36 weeks’ PMA or at discharge from the NICU [30].	<33 weeks	1540 vs 6011	400 + meters vs.86–400 meters	Unadjusted
Rojas, 2012 [31]	Colombia	South America	Case-control	A supplemental oxygen dependency at 36 weeks’ PMA for ≥28 days [8]	27-31 weeks	88 vs. 79 & 45	2600 meters Bogota vs. 959 meters Bucaramanga & 956 meters Cali	Unadjusted
Alshehri, 2014 [32]	Saudi Arabia	Asia	Cross-sectional	A supplemental oxygen dependency at 36 weeks’ PMA for ≥28 days [8]	<32 weeks GA and <1500 gr VLBW	942 vs. 186	2200 meters vs. 650 meters	Unadjusted
Fernandez, 2014 [19]	multiple cities and multiple countries	South America	Cross-sectional	A supplemental oxygen dependency at 36 weeks’ PMA for ≥28 days [8]	≤ 1500 gr	221 vs. 491	2125-2850 meters vs. below 100 meters	Adjusted and unadjusted
Gulliver, 2016 [33]	USA	North America	Cross-sectional	A supplemental oxygen dependency at 36 weeks’ PMA.	23-29 weeks GA	464	1524 meters	Adjusted and unadjusted
Gulliver, 2018 [34]	USA	North America	Cross-sectional	A supplemental oxygen dependency at 36 weeks’ PMA for ≥28 days [8]	23-29 weeks GA	561	1524 meters	Adjusted and unadjusted
Vasquez, 2018 [35]	Colombia	South America	Observational longitudinal	A supplemental oxygen dependency at 36 weeks’ PMA for ≥28 days [36].	≤32 weeks GA	335	2600 meters	Adjusted and unadjusted
Buendia, 2021 [18]	Colombia	South America	Cohort	A supplemental oxygen dependency at 36 weeks’ PMA for ≥28 days [37].	28-31 weeks	223	2200 meters	Adjusted
Han, 2022 [27]	Tibet	Asia	Case-control	A supplemental oxygen dependency at 36 weeks’ PMA for ≥28 days [38].	28-32 weeks GA	124 vs. 227	3600 meters vs. 50 meters	Unadjusted

Abbreviations: BPD – bronchopulmonary dysplasia; GA – gestational age; NICHD - National Institute of Child Health and Human Development;

### The risk of bias assessment

The risk of bias assessment results are presented in Table 2. All the studies had low risk of bias with a CASP score above 7.5.

**Table 2:** CASP risk of bias assessment.

Author, year	Aim	Methodology	Design	Recruitment	Data collection	Researcher-Participant Relationship	Ethical Consideration	Data analysis	Finding	Values	Score
Britton, 2012 [28]	Yes	Yes	Yes	Yes	Yes	No	Yes	Yes	Yes	Yes	9
Lee, 2012 [29]	Yes	Yes	Yes	Yes	Can’t tell	No	Yes	Yes	Yes	Yes	8.5
Rojas, 2012 [31]	Yes	Yes	Yes	Yes	Yes	No	Yes	Yes	Yes	Yes	9
Alshehri, 2014 [32]	Yes	Yes	Yes	Yes	Yes	No	Yes	Yes	Yes	Yes	9
Fernandez, 2014 [19]	Yes	Yes	Yes	Yes	Yes	No	Yes	Yes	Yes	Yes	9
Gulliver, 2016 [33]	Yes	Yes	Yes	Yes	Yes	No	Yes	Yes	Can’t tell	Yes	8.5
Gulliver, 2018 [34]	Yes	Yes	Yes	Yes	Yes	Yes	Yes	Yes	Yes	Yes	10
Vasquez, 2018 [35]	Yes	Yes	Yes	Yes	Yes	Yes	Yes	Yes	Yes	Yes	10
Buendia, 2021 [18]	Yes	Yes	Yes	Yes	Yes	Yes	Yes	Yes	Yes	Yes	10
Han, 2022 [27]	Yes	Yes	Can’t tell	Can’t tell	Can’t tell	Can’t tell	Can’t tell	Yes	Yes	Yes	7.5

Abbreviations: CASP - Critical Appraisal Skills Programme.

### Altitude-unadjusted BPD prevalence rate

**Fig 2:**
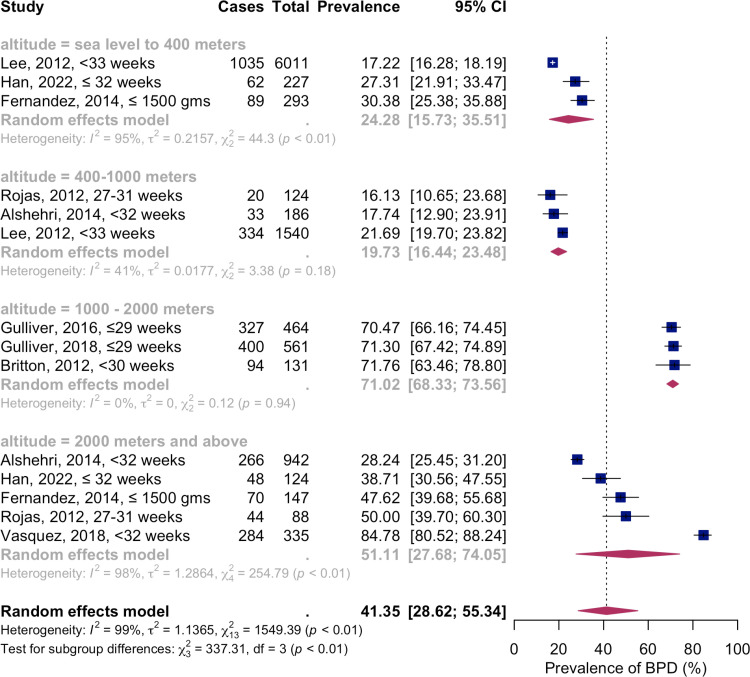
Meta-analysis of the altitude-unadjusted BPD prevalence in preterm and VLBW infants. Abbreviations: BPD – bronchopulmonary dysplasia; CI - confidence interval; VLBW – very low birth weight.

Utilizing a random-effects model, the combined prevalence estimates of altitude-unadjusted BPD from nine studies, constituting fourteen groups, among preterm or VLBW infants, were calculated at 41.35% (3106/11173 participants; [95% CI 28.62; 55.34%]). The substantial heterogeneity was indicated by I^2^=99%, Q (df=13)=1735.55, p-value<0.001 ( Fig 2). In the sea level to 400 meters altitude group, the pooled prevalence of altitude-unadjusted BPD was 24.28% (1186/6531 patients; [95% CI 15.73; 35.51]), demonstrating high heterogeneity (I^2^=95%, Q(df=2)=44.3, p-value<0.01). At 400–1000 meters altitude, the pooled prevalence was 19.73% (387/1850 patients; [95% CI 16.44; 23.48]), with moderate heterogeneity (I^2^=41%, Q(df=2)=3.38, p-value=0.18). At 1000–2000 meters altitude, the prevalence rose sharply to 71.02% (821/1156 patients; [95% CI 68.33; 73.56]), with no heterogeneity (I^2^=0%, Q(df=2)=0.12, p-value=0.94). Meanwhile, at altitudes above 2000 meters, the unadjusted BPD prevalence was 51.11% (712/1636 patients; [95% CI 27.68; 74.05]), with high heterogeneity (I^2^=98%, Q(df=4)=254.79, p-value<0.01).

### Sensitivity analysis

**Fig 3:**
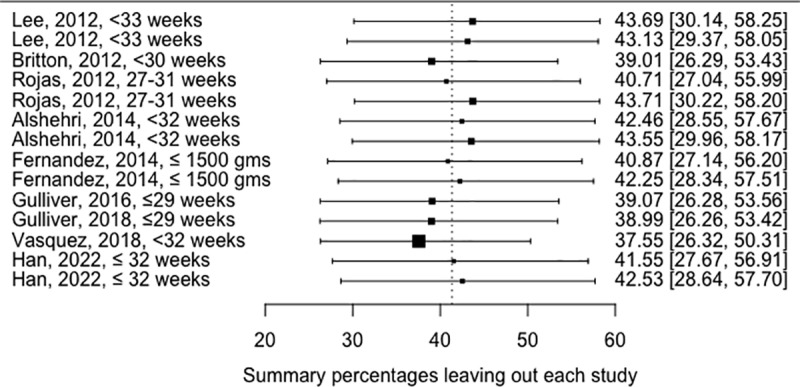
Leave-one-out analysis results.

The leave-one-out study showed that the most influential study is the Vasquez et al., 2018 that reported the highest altitude-unadjusted BPD at 2000 meters and above (Fig 3).

### Publication bias assessment

**Fig 4:**
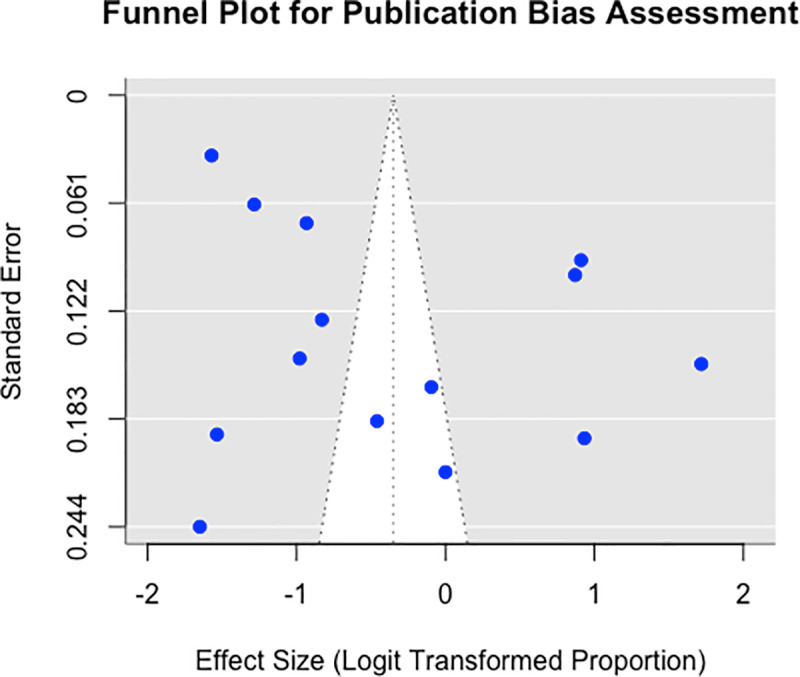
Funnel plot assessing publication bias in meta-analysis of altitude-unadjusted BPD prevalence in preterm and VLBW infants.

Visual inspection of the funnel plot showed no obvious asymmetry (Fig 4), corroborated by Egger’s test (p=0.79), signifying minimal evidence of publication bias in this meta-analysis.

### Altitude-adjusted BPD prevalence rate

**Fig 5:**
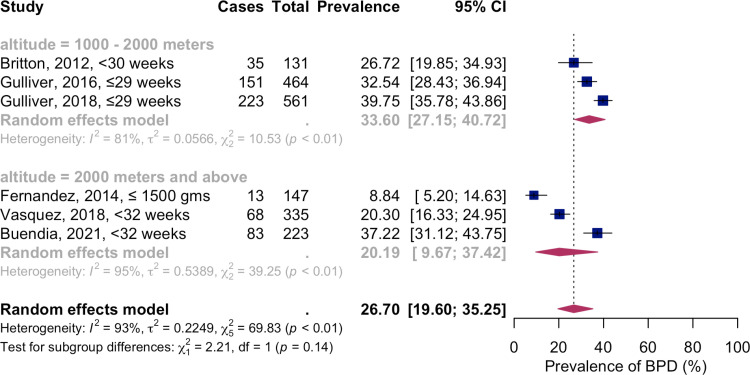
Meta-analysis of altitude-adjusted BPD prevalence in preterm and VLBW infants. Abbreviations: BPD – bronchopulmonary dysplasia; CI - confidence interval; VLBW – very low birth weight.

Using a random-effects model, the combined prevalence of altitude-adjusted BPD among preterm or VLBW infants, based on six studies, was 26.70% (573/1861 participants; [95% CI 19.60; 35.25%]). The high heterogeneity indicated by I^2^=93%, Q (df=5)=69.83, p-value<0.01, reflects variability across the studies (Fig 5). In the altitude range of 1000–2000 meters, the pooled prevalence of altitude-adjusted BPD stood at 33.60% (409/1156 patients; [95% CI 27.15; 40.72]), with significant heterogeneity (I^2^=81%, Q(df=2)=10.53, p-value<0.01). Similarly, at altitudes above 2000 meters, the prevalence was 20.19% (164/705 patients; [95% CI 9.67; 37.42]), also exhibiting high heterogeneity (I^2^=95%, Q(df=2)=39.25, p-value<0.01).

### Sensitivity analysis

**Fig 6:**
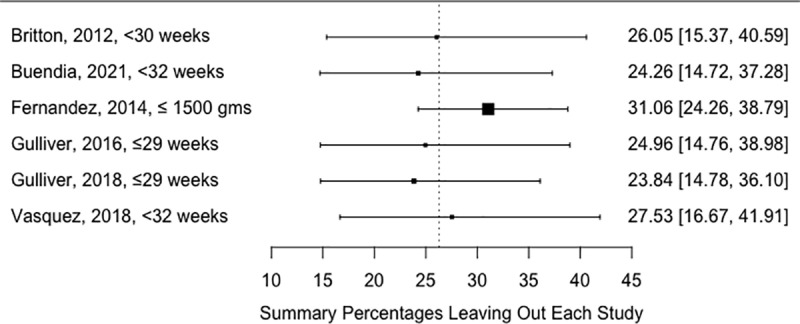
Leave-one-out analysis results.

According to the leave-one-out analysis results, the Fernandez et al., 2014, reporting the lowest altitude-adjusted BPD at 2000 meters and above, emerged as the most influential study (Fig 6).

### Certainty of evidence assessment

Based on the GRADE certainty assessment (Table 3), both the pooled altitude-adjusted and altitude-unadjusted BPD prevalence rates have a low certainty of evidence and should be interpreted with caution. Furthermore, in this meta-analysis, the first analysis included 14 studies, allowing for a valid publication bias assessment, while the second analysis included only seven studies, making publication bias assessment unreliable for altitude-adjusted BPD prevalence rate.

**Table 3:** GRADE certainty of evidence assessment.

Outcome	Study Design	Risk of Bias	Inconsistency	Indirectness	Imprecision	Publication Bias	Certainty of Evidence
Altitude-unadjusted BPD prevalence	Meta-analysis of observational studies	Low	Serious	Not serious	Not serious	Undetected	Low
Altitude-adjusted BPD prevalence	Meta-analysis of observational studies	Low	Serious	Not serious	Not serious	None	Low

Abbreviations: GRADE - Grading of Recommendations Assessment, Development, and Evaluation.

## Discussion

The systematic review and meta-analysis investigated the prevalence of BPD among preterm and VLBW infants, considering variations in altitude. The study found substantial variations in BPD prevalence across different altitudes when the altitude was not adjusted. Interestingly, studies that reported the BPD prevalence at 400–1000 meters and at 1000–2000 meters were homogenous. Not surprisingly, higher altitudes correlated with increased BPD prevalence, with the most significant rates observed at altitudes 1000–2000 meters, followed by 2000 meters and above.

Altitude adjustment proved crucial for accurately assessing the prevalence of BPD. While unadjusted rates indicated a higher prevalence at greater altitudes, adjustment mitigated this prevalence, though with notable heterogeneity. Furthermore, adjusted BPD prevalence remained higher at altitudes ranging from 1000 to 2000 meters compared to those above 2000 meters, averaging at 26.70%. The available literature on this matter presents conflicting findings. Particularly, our altitude-adjusted rate notably differs from the 46% reported in a validation study of the National Institute of Child Health and Human Development (NICHD) definition for moderate and severe BPD [39]. Moreover, a recent systematic review revealed a wide range of global BPD incidence, from 17% to 75%, at 36 corrected postmenopausal age, mirroring our findings in the altitude-unadjusted BPD prevalence analysis [40]. The authors of this study speculate that altitude variability contributes, at least in part, to the observed variations in global BPD prevalence.

Our findings underscore the complex interplay between altitude and BPD prevalence, highlighting the importance of considering geographical factors in assessing respiratory outcomes among preterm infants. The included studies revealed a notable variation in BPD prevalence across different altitude ranges, with distinct patterns emerging at varying altitudes. Previously, Pagani et al., 2017 emphasized the multifactorial etiology of BPD, implicating neuronal, mechanical, and autonomic factors in airway control during early childhood [41]. This suggests that altitude-related physiological changes, such as variations in oxygen partial pressure, may interact with underlying respiratory vulnerabilities to influence BPD risk.

Grant and colleagues performed a comprehensive systematic review and meta-analysis to investigate the relationship between the altitude and probability of adverse perinatal outcomes, such as low birth weight (LBW), small for gestational age (SGA), and spontaneous preterm birth (sPTB) [42]. Their findings revealed a significant correlation between elevated altitudes and these adverse outcomes. This underscores the importance of factoring altitude into the management strategies for preterm and VLBW infants.

Limitations of the present analysis include the exclusion of non-English language publications, which may introduce language bias. To mitigate this limitation, future research should include non-English studies to minimize language bias and improve generalizability. By broadening the scope of the search, researchers can minimize bias and improve the generalizability of findings to diverse linguistic contexts. Additionally, the scarcity of included literature from South and East Asia presents another limitation. Specifically, the analysis incorporated only one study with data from Tibet, with no information on other high-altitude locations in the region. This paucity of data underscores the need for further investigations to fill this gap and provide a more comprehensive understanding of the relationship between altitude and BPD across diverse geographical regions. A major limitation is the high heterogeneity among included studies, contributing to the low certainty of evidence. This heterogeneity may stem from variations in study design, population characteristics, diagnostic criteria for BPD, and altitude correction methodologies. To better understand the sources of heterogeneity, future meta-analyses could consider conducting subgroup analyses based on factors such as region, gestational age, or birth weight. Such analyses may help identify specific subgroups or conditions under which the relationship between altitude and BPD is more pronounced or consistent. Finally, it is important to acknowledge the limitations of using oxygen support at 36 weeks PMA as the sole criterion for diagnosing BPD that we have used in our study. Recent evidence-based approaches, such as the definition proposed by Jensen et al., 2019, emphasize the use of respiratory support levels rather than oxygen dependence to classify BPD severity [36]. Future studies should consider incorporating such updated diagnostic criteria to enhance the accuracy and relevance of BPD prevalence estimates, particularly in high-altitude regions where respiratory support needs may differ.

Practical Implications: Understanding altitude-related variations in BPD prevalence is essential for clinicians and policymakers. Our findings demonstrate that altitude is significantly associated with the risk of BPD among preterm and VLBW infants, with higher altitudes (1000–2000 meters and above) associated with a marked increase in BPD prevalence. The increased prevalence of BPD at higher altitudes is not merely a subjective observation but reflects the compounded impact of hypoxia, oxidative stress, and impaired lung development in these vulnerable populations. As Domm and colleagues note, adapting to low oxygen levels at birth can result in maladaptive health effects in children, akin to those caused by high oxygen exposure [43].

Clinically, these findings underscore the need for tailored respiratory support strategies in high-altitude regions. Furthermore, antenatal counseling for expectant mothers in high-altitude regions should include discussions about the increased risk of BPD and the potential need for specialized neonatal care. From a public health perspective, our results highlight the importance of altitude adjustment in epidemiological studies to ensure accurate prevalence estimates and equitable resource allocation.

In conclusion, this systematic review and meta-analysis provide valuable insights into altitude-related variations in BPD prevalence among preterm and VLBW infants. The findings underscore the need for standardized reporting of altitude levels and the inclusion of altitude as a covariate in epidemiological research and healthcare planning to address the specific needs of infants in high-altitude regions.

## Supporting information

S1 Table PRISMA checklist for systematic reviews. (DOCX)

S2 Table Study selection process results. (XLSX)

S3 Table Comprehensive data extraction details. (XLSX)
